# A Source-Initiated On-Demand Routing Algorithm Based on the Thorup-Zwick Theory for Mobile Wireless Sensor Networks

**DOI:** 10.1155/2013/283852

**Published:** 2013-12-25

**Authors:** Yuxin Mao, Ping Zhu

**Affiliations:** School of Computer and Information Engineering, Zhejiang Gongshang University, Xuezheng Street No. 18, Hangzhou, Zhejiang 310018, China

## Abstract

The unreliability and dynamics of mobile wireless sensor networks make it hard to perform end-to-end communications. This paper presents a novel source-initiated on-demand routing mechanism for efficient data transmission in mobile wireless sensor networks. It explores the Thorup-Zwick theory to achieve source-initiated on-demand routing with time efficiency. It is able to find out shortest routing path between source and target in a network and transfer data in linear time. The algorithm is easy to be implemented and performed in resource-constrained mobile wireless sensor networks. We also evaluate the approach by analyzing its cost in detail. It can be seen that the approach is efficient to support data transmission in mobile wireless sensor networks.

## 1. Introduction

As a kind of wireless technology, wireless sensor networks (WSNs) [1, 2] are systems that comprise large numbers (usually hundreds or thousands) of wirelessly connected heterogeneous sensor nodes that are spatially distributed across a large field of interest. There is a wide range of applications where the WSNs are extensively used, and their development in other applications is still growing. Mobile wireless sensor networks (MWSNs) are a particular class of WSN in which mobility plays a key role in the execution of the application. In many cases, MWSNs suffer from link breakages and frequent changes of network topology. For example, a sensor node with a limited battery life may sleep periodically in order to reserve energy. A sensor node may also be blocked by data packets from its neighbours at some time or jammed by malicious nodes. Hence, a normal sensor node will lead to denial of service in those situations. Moreover, intermediate nodes are often required to carry out end-to-end communications since the transmission range of sensor nodes is also limited. Therefore, the intrinsic features of MWSNs make it hard to perform end-to-end communications, especially for large-scale data transmission [3, 4].

In this paper, we propose a novel approach of source-initiated on-demand routing [5–7] for MWSNs. We explore the Thorup-Zwick theory [8] to achieve efficient end-to-end communications in MWSNs. The remaining of the paper is organized as follows.  Section 2 illustrates the network model and problem statement for the approach. In Section 3, we present an efficient algorithm for source-initiated on-demand routing in MWSNs. We evaluate the algorithm from the point of cost and complexity in Section 4 and discuss the simulation results in detail.  Section 5 gives an overview of the related works.  Section 6 concludes the paper with an outlook to future research directions.

## 2. Network Model and Problem Statement

**Figure 1:**
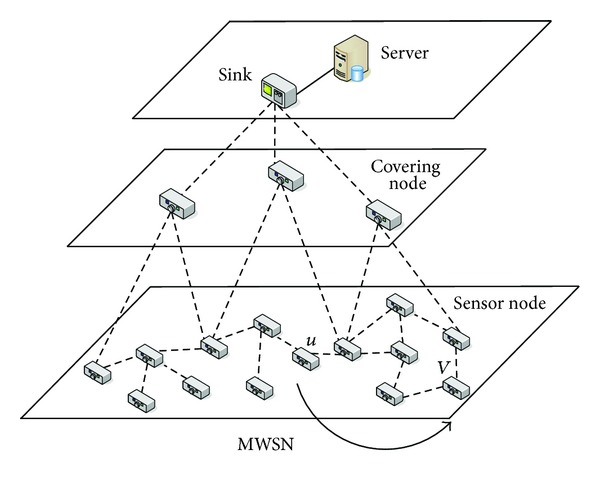
The three-tier network structure for MWSN.

In this paper, we consider a relatively simple MWSN model. *I* = {1, 2, …, *n*} and assume a MWSN with *k* nodes. We assume the whole network consists of three tiers (see Figure 1). In the bottom, there are a number of sensor nodes. Each node has a unique identity *i*  (*i* ∈ *I*) in the network. Each node in the network is battery-powered and has limited computation and wireless communication capabilities. We assume that the locations of the sensor nodes are relatively static, rather than moving. Without confusion, we will also use *n*_*i*_ to denote the location of a sensor node *n*_*i*_, *i* ∈ *I*.

There is a sink node in the top level. We assume that the sink is a center equipped with sufficient computation and storage capabilities. Although the sink is able to communicate with each sensor node directly, direct communication between the sink and a sensor node is time consuming and energy consuming. For example, if a sensor node sends a large file (e.g., video file) to the sink, the energy of the sensor node will soon be exhausted. Therefore, we will decrease this kind of direct communication. Instead, the sink will allocate information from the covering nodes timely.

There are several *covering nodes* in the middle level, which are similar with cluster heads in clustering hierarchy or relay nodes in flat hierarchy. These nodes are only used to collect status information from sensor nodes, without any further processing or computation. Each covering node covers a part of the network with a number of sensor nodes. The placement of the covering nodes will ensure that all the sensor nodes in the network are covered. Also we will ensure that there are no more than *η* hops between a covering node and a sensor node that it covers (usually *η* ≤ 3). Covering nodes are able to communicate with the sink directly.

Let *x* and *y* be two points in the Euclidean plane, then  [*x*, *y*]  denotes the line segment connecting *x* and  *y*, and  |*x*, *y* | denotes the Euclidean distance between *x* and *y*. Two sensor nodes *n*_*i*_ and *n*_*j*_ can communicate with each other if |*n*_*i*_, *n*_*j*_| < *R*, where *R* is the communication range of a sensor node in the MWSN.

The major task of a sensor node in the network is to communicate with other nodes and transmit data to others by routing. As we have mentioned before, intermediate nodes are often required to carry out end-to-end communications. Therefore, routing in this network should provide a *path* from source to destination and the path itself should be as short as possible. In this work, we do not consider the situation when there are selfish nodes in the network. We assume that each node is willing to cooperate with its neighbours.

## 3. Source-Initiated On-Demand Routing Algorithm

We attempt to find a short path for a source node in a MWSN by using some routing algorithm. The major difficulty of designing the routing algorithm is the cost in path construction. Due to the link breakages and frequent changes of network topology, a source node has to update its routing paths frequently, which is obviously time consuming. The situation becomes worse when the network grows up in size. In this work, we explore the Thorup-Zwick theory to solve the problem and achieve efficient routing in MWSNs.

### 3.1. Algorithm Overview

An overview of the source-initiated on-demand routing algorithm is given in Algorithm 1. Given a time *t*, the network topology of a MWSN can be denoted by a weighted undirected graph *G* = (*V*, *E*). *V* is the sets of sensor nodes in the network. *E* is the sets of connection status among the nodes. When node  *u* attempts to send data to node *v*, it first checks its local cache. If there is no existing routing path between them, *u* will send a routing request to the sink (*s*). Then the sink will query its local database that contains the data structure. Here the data structure is generated by preprocessing *G*. The sink will send back a notification to *u*, which contains the shortest path from *u* to *v*. Finally, *u* sends data to *v* by using the feedback path.

The algorithm is straightforward. The key point is how to perform path query from *u* to *v*. We will give the detailed explanation in Sections 3.3 and 3.4.

### 3.2. Status Allocation

As we have mentioned in Section 2, sensor nodes are not encouraged to communicate with the sink directly. However, the sink requires some basic information from sensor nodes in order to support source-initiated on-demand routing. This information contains the location of a sensor node as well as its connection status currently. The algorithm for this process of status allocation is illustrated in Algorithm 2.

The algorithm in Algorithm 2 is trivial. Each sensor sends a status vector to its covering node. The status vector contains the factors that have impacts on data communication. The status vector of a sensor node *n*_*i*_ can be formally represented by *v*_*i*_ = 〈*E*_*i*_, PRR_*i*_, *L*_*i*_, *C*_*i*_〉, where *E*_*i*_ is the value of available energy of *n*_*i*_, PRR_*i*_ denotes the packet reception ratio (PRR) at *n*_*i*_, which is a metric for evaluating link quality, *L*_*i*_ is the load of *n*_*i*_, and *C*_*i*_ denotes the connection status of *n*_*i*_ (the direct neighbors of the node).

For each window of Δ*w* received packets at *n*_*i*_, PRR_*i*_ is computed as follows:

(1) $$\begin{array}{c} {\text{PRR}}_{i}\left(\Delta w\right)=\frac{{\text{Num}}_{\text{rp}}}{{\text{Num}}_{\text{sp}}}, \end{array}$$

where Num_rp_ denotes the number of successfully received packets, while Num_sp_ the number of transmitted packets. For a given timeframe Δ*t*, the load *L*_*i*_ is computed as follows:

(2) $$\begin{array}{c} L_{i}\left(\Delta t\right)=\frac{\text{Nu}{\text{m}}_{\text{rdp}}}{\text{Nu}{\text{m}}_{\text{lgp}}}, \end{array}$$

where   Num_rdp_ denotes the number of relayed data packets (not locally generated), while Num_lgp_ the number of locally generated packets.

After collecting the information, the covering node then forwards it to the sink together.

**Figure 2:**
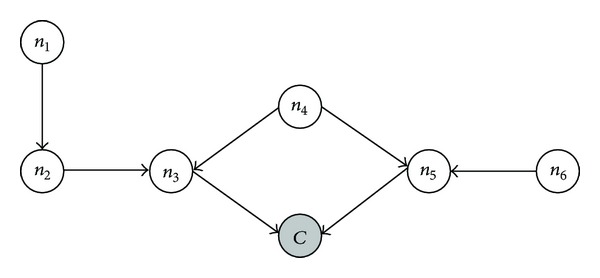
An example of status allocation by covering node.

Moreover, there are different cases for status allocation in a MWSN. If we set *η* to 3, there are three kinds of cases for status allocation. Take the subnetwork shown in Figure 2 for example: (a) to the sensor nodes (*n*_3_ and *n*_5_) directly adjacent to a covering node (*C*), they are able to send status vectors to it. (b) To the sensor nodes (*n*_2_, *n*_4_ and *n*_6_) directly adjacent to the ones in the first case, they could send status vectors to the covering node by two hops. (c) To the rest of the sensor nodes (*n*_1_), they have to send status vectors by three hops.

### 3.3. Graph Construction

After allocating status from distributed sensor nodes, the sink is able to get the overall information of the network. Given a time  *t*, the network topology of a MWSN can be denoted by a weighted undirected graph  *G* = (*V*, *E*). Assume  |*V* | = *n*  and  |*E* | = *m*. Each element in *V* denotes a sensor node in the MWSN and each element in *E* denotes a link between two nodes. For all *e*_*ij*_ ∈ *E*, we have the following equation:

(3) $$\begin{array}{c} e_{ij}=\{\begin{array}{cc} w_{ij}\cdot \left|n_{i},n_{j}\right|, & \left|n_{i},n_{j}\right|<R,\\ \infty , & \left|n_{i},n_{j}\right|\geq R. \end{array} \end{array}$$

It means that the distance between any two nodes in the graph is a weighted value. If the Euclidean distance between two nodes is greater than the communication rage  *R*, we just set the distance value to be *∞* in the graph. The key to the graph construction is to fix the weight values for each edge in the graph. Weight is formally defined as follows:

(4) $$\begin{array}{c} w_{ij}=\alpha \cdot E_{ij}+\beta \cdot Q_{ij}+\gamma \cdot L_{ij}. \end{array}$$

The weight depends on several factors. *E*_*ij*_ denotes the energy status for the two nodes. The value of *E*_*ij*_ is calculated by

(5) $$\begin{array}{c} E_{ij}=\frac{E_{i}-E_{t}}{E_{i}}\times \frac{E_{j}-E_{t}}{E_{j}}, \end{array}$$

where *E*_*i*_ and *E*_*j*_ are the values of available energy for *n*_*i*_ and *n*_*j*_ and *E*_*t*_ is the energy required for an operation of data transmission. *Q*_*ij*_ denotes the link quality between *n*_*i*_ and *n*_*j*_. Here we try to use software-based link quality estimators [9–13] to evaluate the link quality. We integrate the ETX estimator [14] to get an estimate of the link quality. *Q*_*ij*_ is calculated as follows:

(6) $$\begin{array}{c} Q_{ij}=\frac{1}{{\text{PRR}}_{i}\times {\text{PRR}}_{j}}, \end{array}$$

where PRR_*i*_ reflects the uplink quality from *n*_*i*_ to *n*_*j*_, while PRR_*j*_ the downlink quality from *n*_*j*_ to *n*_*i*_. *L*_*ij*_ denotes the load status for the two nodes. The value of *L*_*ij*_ is calculated by

(7) $$\begin{array}{c} L_{ij}=\frac{1}{L_{i}\times L_{j}}. \end{array}$$

*α*,  *β*, and *γ* are coefficients for the weight and we have *α* + *β* + *γ* = 1.

### 3.4. Graph Preprocessing

In order to perform efficient path query in the graph for a MWSN, we need to preprocess the weighted undirected graph *G* first. Assume  |*V* | = *n*  and  |*E* | = *m*. Thorup and Zwick in [8] have proposed an approach of preprocessing *G* in *O*(*knm* ^1/*k*^) expected time and constructing a data structure with size *O*(*kn* ^1+1/*k*^). Any subsequent path query can be answered approximately in *O*(*k*) time. The approximate distance returned is of stretch at most 2*k* − 1. Here *k* is an integer and *k* ≥ 1. After allocating status information, the sink is able to get the topology of the MWSN. Therefore, we could use the Thorup-Zwick theory directly to preprocess the graph structure of the MWSN (see Algorithm 3).

### 3.5. Path Query

After preprocessing the graph structure of the MWSN, the sink is able to answer a path query in linear time. The algorithm of path query is given in Algorithm 4. Here we make use of the Thorup-Zwick theory to perform path query in the database structure returned by the preprocessing algorithm.

## 4. Evaluation

In this section, we mainly evaluate the performance of the proposed algorithm by analyzing its complexity and cost, against existing routing algorithms for MWSNs.

The cost for the proposed algorithm is mainly generated from four activities: status allocation, graph construction, graph preprocessing, and path query. The first three activities are preprocessing ones. We try to evaluate the cost for these four activities by analyzing the time complexity.

We evaluate the cost for status allocation at first. According to Section 3.2, we have the following equation:

(8) $$\begin{array}{c} n=N_{1}+N_{2}+N_{3}. \end{array}$$

Here *N*_1_, *N*_2_, and *N*_3_ denote the number of sensor nodes in the three cases in status allocation, respectively. Assume that the one hop (sensor node to sensor node or sensor node to covering node) cost for status allocation is Δ*c*_1_, and the one hop between covering node and sink is Δ*c*_2_. Then the total cost Δ*C* for status allocation is as follows:

(9) $$\begin{array}{c} \Delta C=N_{1}\Delta c_{1}+N_{2}2\Delta c_{1}+N_{3}3\Delta c_{1}+n\Delta c_{2}. \end{array}$$

We could reduce (9) into

(10) $$\begin{array}{c} \Delta C=\left(2\Delta c_{1}+\Delta c_{2}\right)n+\Delta c_{1}\left(N_{3}-N_{1}\right). \end{array}$$

As |*N*_3_ − *N*_1_| < *n*, then we have:

(11) ΔC=(2Δc1+Δc2)n+Δc1(N3−N1)<(2Δc1+Δc2)n+Δc1n=(3Δc1+Δc2)n.

Therefore, the cost for status allocation is  *O*(*n*).

We have to compute the weight for each edge in graph construction. Therefore, the cost for graph construction is  *O*(*m*). According to [8], the cost for graph preprocessing is *O*(*knm* ^1/*k*^) and the approximate cost for answering path query is  *O*(*k*). Finally, we can get the preprocessing cost as *O*(*n*) + *O*(*n*) + *O*(*m*) + *O*(*knm* ^1/*k*^) = *O*(2*n* + *m* + *knm* ^1/*k*^) = *O*(*n* + *m* + *knm* ^1/*k*^) and the query cost as  *O*(*k*). We can see that the activities in our algorithm have linear cost except graph preprocessing. If we set *k* to be a large integer, then the cost for graph preprocessing is also not very high and acceptable to MWSNs.

## 5. Related Works

Generally, existing routing protocols for WSNs fall into two categories: table-driven and on-demand routing [7] based on when and how the routes are discovered. For the table-driven routing protocols, consistent and up-to-date routing information for all the sensor nodes are maintained at each mobile host. It has been shown in [15] and stated in [16] that on-demand routing protocols can perform better than table-driven protocols in WSNs.

There have been many on-going research efforts in on-demand routing for WSNs or wireless networks. For example, the ad hoc on-demand distance vector routing (AODV) [17] is an improvement of the destination-sequenced distance-vector (DSDV) algorithm. AODV minimizes the number of broadcasts by creating routes on-demand as opposed to the DSDV that maintains a list of all the routes. The dynamic source routing protocol (DSR) [18] is another on-demand routing protocol. A sensor node maintains the route caches containing the source routes that it is aware of. The mobile host updates the entries in the route cache as soon as it learns about new routes. The temporally ordered routing algorithm (TORA) [19] is a highly adaptive, efficient, and scalable distributed routing algorithm based on the concept of link reversal. TORA is proposed for highly dynamic mobile and multihop wireless networks. It is a source-initiated on-demand routing protocol. However, none of these algorithms take the case that how to perform efficient routing in a WSN with link breakages and frequent changes of network topology into consideration.

Some routing algorithms just enhance the abovementioned ones with fault-tolerant or energy-balancing mechanism [20–23]. However, these algorithms are not able to provide short path for routing or they do not provide an efficient way for constructing short path. Moreover, there are also a few algorithms about shortest path routing [24, 25]; however, these algorithms fall short of efficiency due to high cost or large complexity.

Compared with existing works in this field, our approach uses a novel graph-based mechanism that makes full use of the Thorup-Zwick theory to improve the end-to-end communication in MWSNs. The algorithm is of time efficiency and the overhead is acceptable to large-scale MWSNs. The advantage of our approach is that we can still achieve efficient routing even the number of the nodes in a MWSN grows up.

## 6. Conclusion

In this study, we mainly present a novel source-initiated on-demand routing algorithm for efficient data transmission in MWSNs. We explore the Thorup-Zwick theory to achieve source-initiated on-demand routing with time efficiency. With this algorithm, we are able to find out shortest routing path between source and target in a network and transfer data in linear time. The algorithm is also easy to be implemented and performed in resource-constrained MWSNs. We also evaluate the algorithm by analyzing its time complexity in detail. It can be seen that the approach is efficient to support end-to-end data communication in MWSNs. Compared with existing works in this field, our approach is of time efficiency and the overhead is acceptable to large-scale MWSNs. The advantage of our approach is that we can still achieve efficient routing even the number of the nodes in a MWSN grows up.

Future works may include: (1) improving the efficiency of the algorithms to reduce the operations of graph preprocessing; (2) considering a more complex MWSN model to implement and evaluate the approach; and (3) considering the security problem of routing in MWSNs.

## Figures and Tables

**Algorithm 1:**
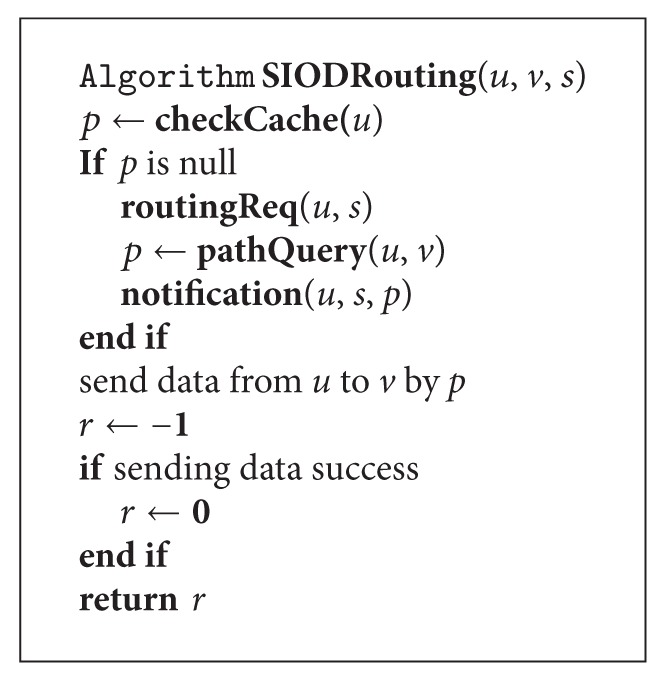
The source-initiated on-demand routing algorithm for MWSNs.

**Algorithm 2:**
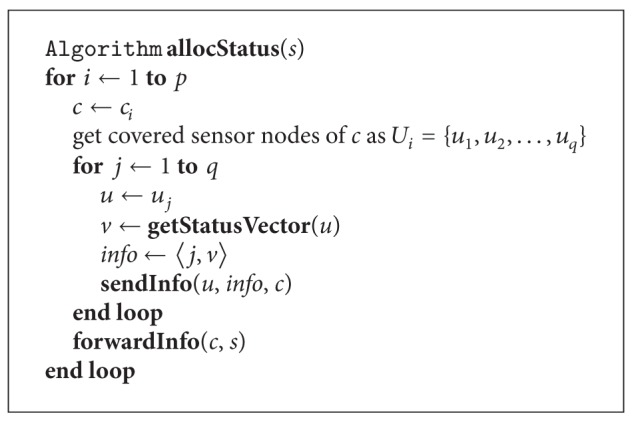
Allocating status information from sensor nodes via covering nodes.

**Algorithm 3:**
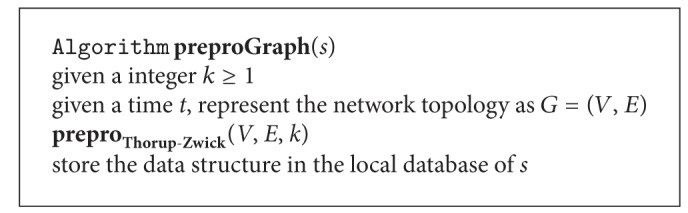
Preprocessing the graph structure of a MWSN.

**Algorithm 4:**
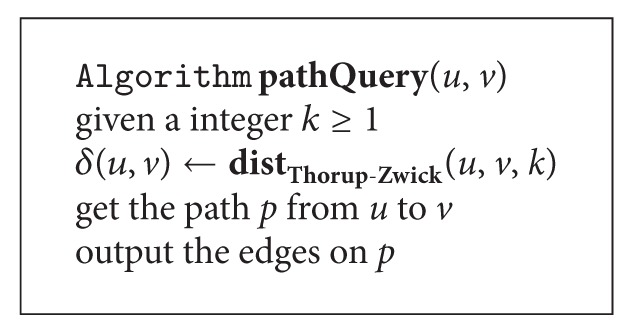
Path query on the graph structure of a MWSN.
